# Effect of Creatine Monohydrate on Spatial Working Memory, Body Weight, and Food Intake in Male and Female Rats

**DOI:** 10.3390/nu17132218

**Published:** 2025-07-04

**Authors:** Cyrilla Wideman, Alexandria Iemma, Olivia Janolo, Anastasiya Kalinina, Helen Murphy

**Affiliations:** Interdisciplinary Neuroscience Concentration, John Carroll University, University Heights, OH 44118, USAhmurphy@jcu.edu (H.M.)

**Keywords:** rat, Morris water maze, creatine supplement, spatial working memory, gender

## Abstract

**Background/Objectives:** Creatine monohydrate supplementation has gained popularity in the fitness industry due to its ability to enhance athletic performance and has sparked curiosity about other possible effects of the supplement. The purpose of this study was to examine the effect of creatine supplementation on spatial working memory, body weight, and food intake in male and female rats. **Methods:** Experimental rats, six male and six female, were administered creatine while six male and six female rats served as controls. The Morris water maze (MWM) was employed to assess spatial working memory. Body weight and food intake were measured daily. **Results:** Neither male control nor experimental animals demonstrated positive working memory upon initial exposure (week 1) to the MWM, whereas the initial exposure of female control and experimental animals resulted in positive working memory. By week 2 of the experimental period, all animals in both the control and experimental groups showed significant working memory with no significant differences among the groups. These effects were unrelated to creatine supplementation. Gender-specific differences were found for body weight, with higher weight gain observed in male rats compared to female rats. Weight gain was not directly influenced by creatine supplementation; however, food intake was lower in the experimental male rats receiving the supplement as compared to the control rats. No difference was observed in female rats. **Conclusions:** Because of the popularity of creatine, further research about the effects of this supplement on different mechanisms in the body influencing cognitive processing and appetitive behavior is warranted.

## 1. Introduction

Creatine is a naturally occurring compound that provides immediate energy to areas of high energy demand, such as the muscles and brain. The compound is produced by the amino acids, glycine, arginine, and methionine in the liver, kidneys, and pancreas. After production, creatine kinase converts creatine into the energy source phosphocreatine, which is primarily stored in the muscles of the body to be used as energy. The high-energy nature of the phosphate bonds in phosphocreatine allows the compound to be readily available for rapid ATP replacement in energetically demanding situations [1]. While the body naturally produces creatine, the compound can be acquired in other ways, such as through the consumption of seafood and red meat, which provide far less creatine than synthetic sources [2]. 

The way that creatine acts as an energy source to muscle tissue has created a surge in popularity in the fitness industry. Creatine has been widely supplemented by athletes participating in highly intensive activities in order to improve their performance and achieve more rapid recovery during training and competition [1]. Athletes that implement dietary supplementation of creatine typically start with a loading dose of 20 g of creatine per day for a week. This loading dose serves as a way for the muscles to be saturated by the creatine much faster, which speeds up the effects of creatine on the body. Following the loading dose, athletes continue to use a maintenance dose of 5 g per day for the rest of the supplementation period [3]. While creatine comes in many forms, creatine monohydrate is the most common form used in supplementation and has been shown to increase athletic performance in humans. Creatine monohydrate can be administered orally, since its powder form can be dissolved in water or juice and the efficacy, affordability, and safety of the supplement allows this form to be widely used [4]. The beneficial effects of creatine on physical activity have sparked interest in the effects of creatine on cognition. Similarly to muscle cells, neurons participate in energy-demanding activities [1]. There are, however, conflicting results regarding whether creatine supplementation can improve cognitive function [5,6,7].

While creatine supplementation has been studied extensively in humans, few animal studies have been conducted. Animal research can be more beneficial than human research because of ethical considerations; the ability to control variables such as the environment, circadian cycles, and diet; as well as standardized experimental designs and cognitive tasks. The limited research on animal subjects has yielded similar results to human-focused research in terms of physical performance. Rat studies have shown that creatine has beneficial effects in numerous types of athletic activity, such as swimming [8] and running [9]. The few reports that have examined the effects of creatine supplementation and cognition have paired creatine supplementation with other variables. For example, researchers administered L-arginine, an amino acid necessary for making proteins, with creatine to rats to ascertain its effects on spatial memory [10]. Using the addition of creatine as a control group in this study, it was demonstrated that the supplement had a positive effect on the performance of spatial working memory tasks. When both L-arginine and creatine were co-administered, reference memory was enhanced. Furthermore, a study by Robinson et al. [11] explored the effects of creatine supplementation paired with guanidino compounds on spatial memory in pigs. The specific guanidino compound used in that research was guanidinoacetic acid, which can be transformed into creatine by methylation. The areas by which the guanidino compounds act include the cerebellum and prefrontal cortex, which are areas where some memory consolidation occurs. The results of this experiment showed that creatine metabolism may be crucial in these brain areas to support proper brain function and memory consolidation, but neither supplemental creatine nor guanidinoacetic acid improved memory performance in young pigs.

Because of the paucity of studies conducted on the effects of creatine on spatial working memory in rats, the primary focus of the present study was to investigate this specific area of cognition utilizing the Morris water maze (MWM). It has been demonstrated that the MWM exhibits specificity for hippocampal-dependent learning and memory [12], which is essential due to the involvement of the hippocampus in working memory [13]. In the current experiment, four groups of rats were studied. The control male and female groups received a condensed milk treat serving as a placebo, while the experimental male and female groups received a condensed milk treat containing creatine monohydrate.

Although the primary aim of the current study was to analyze the effects of creatine supplementation on spatial working memory within genders, an additional goal was to measure body weight and food intake both across and between genders. Limited research has explored the possible similarities and differences in the effects of creatine in male and female rats. The findings of Galbraith et al. [14] suggest that creatine concentrations in the brain may play a role in regulating food intake and body weight. Dunham et al. [15] reported that dietary intervention involving creatine monohydrate supplementation affected male and female rats differently. 

Aware of the beneficial effects of creatine on physical activity, we hypothesized that when compared to a control group, creatine-treated rats of both genders would show significantly lower test trial times in the MWM, indicating improved spatial working memory. Furthermore, we hypothesized that creatine-treated animals would have a significantly higher body weight and food intake compared to the male and female control groups. Also, the male rats were predicted to weigh more and have greater appetites than the female rats. 

## 2. Materials and Methods

### 2.1. Animal Housing and Care

All procedures were approved by the local Institutional Animal Care and Use Committee (Protocol #2101d) and followed NIH guidelines. Twelve male Long Evans rats and twelve female Long Evans rats (Envigo, Indianapolis, IN, USA), weighing 80–110 g and approximately four weeks of age, were placed in individual cages that were equipped with a running wheel. The room temperature was maintained between 21 and 23 °C and the 24 h circadian cycle was divided into a 12 h light period followed by a 12 h dark period. Body weight and food consumption were recorded daily. 

### 2.2. Habituation Period

Animals were habituated for one week. At the beginning of the dark cycle, the rats were weighed and given a “treat”: 450 μL of condensed milk in a shallow glass dish. The treat was completely consumed within five minutes. The rats had ad libitum access to Lab Diet Rodent Chow 5001 (Lab Diet, Richmond, IN, USA) and water.

### 2.3. Supplement Administration

Following the habituation period, six male and six female rats were randomly selected by weight to be part of the experimental group while the other six rats from each gender were designated as the control group. In the condensed milk treat, the experimental group received a 300 mg/kg body weight dose of creatine monohydrate (Sigma Aldrich Inc., St. Louis, MO, USA) as utilized by Young and Young [16]. This dosage served as a loading dose that was given for the first five days of the experimental period. Following the loading phase, the rats were given a 75 mg/kg maintenance dose for the remainder of the experimental period [17]. The control group received the treat without creatine, acting as a placebo. Administration of the supplement and placebo to the respective groups was provided to the rats at the start of the dark cycle every day throughout the experimental period.

### 2.4. Morris Water Maze Test

During the three-week experimental period, both groups of rats were assessed once a week using the MWM. The maze was a circular pool that was 1.5 m in diameter and 60 cm deep. It was partitioned into 8 segments and a towel, which served as a cue for the rats, was draped over the side in a permanent location throughout the experiment. The permanent location of the towel kept the rats from associating platform placement with the cue only. Such an association would have introduced bias into the results because, instead of memorizing the location of the platform itself, the rat could learn to associate the location of the cue with the platform. The water in the pool was maintained at room temperature. All maze assessments were conducted utilizing a red-light bulb of 5 lux during the dark cycle, which is the active period in the circadian cycle in rats, an important variable that has not been considered in other studies.

At the end of each week, the general protocol for spatial working memory in rats proposed by Vorhees and Williams [12] was employed for both experimental and control groups. Using a modification of this protocol, the rats were assessed immediately following the consumption of the condensed milk treats [18]. The first run served as a sample trial which consisted of the rat discovering the location of the platform without being familiarized with the maze beforehand. The second run served as a test trial which consisted of the rat recalling the location of the platform. During the sample trial, each rat was given 90 s to find the platform. Once the platform was discovered by the rat, it rested on the platform for 15 s. If the rat did not find the platform after the allotted 90 s, it was guided to it. After 15 s of rest on the platform, the animal was placed back in the start location of the maze. During the test trial, 90 s were allotted for the rat to find the platform. If the rat recalled the location of the platform, it swam faster to the platform during the test trial than the sample trial. After the maze assessment, each rat was patted gently with a paper towel to remove excess water, and then returned to its home cage. Platform location varied in every MWM assessment of the study by following a chart proposed by Vorhees and Williams [12]. Due to platform relocation every week, no learning of the platform position from the previous week transferred to the test trial of the next week; hence, recall on the day of each trial was dependent on the sample trial of that particular day and measured only working memory. 

### 2.5. Statistical Analysis

Data were statistically analyzed using SPSS statistical analysis software (version 29.0.2.0; IBM Corp., Armonk, NY, USA) and R Studio (version 2024.04.2). The equality of variances across groups was checked using Levene’s test. In cases where Levene’s test was violated, *p*-values and 97.5% confidence interval estimates were calculated by bootstrapping from 1000 mixed-design Analysis of Variance (ANOVA) tests to reduce making a Type 1 error, mitigate small sample size issues, and produce more robust confidence intervals. Pairwise *t*-tests were run to identify any significant differences between genders (female vs. male), and treatments (control vs. experimental) by mean run times (sample vs. test) for each of the three experimental weeks. *p*-values were adjusted using Bonferroni correction.

Tukey’s HSD post-hoc pairwise test was used to compare mean food intake by gender and treatment groups in the final experimental week. Similarly, mean body weight was also compared by gender and treatment groups in the final experimental week. A *p*-value of <0.05 was considered significant for all statistical analyses.

## 3. Results

### 3.1. Morris Water Maze

**Figure 1:**
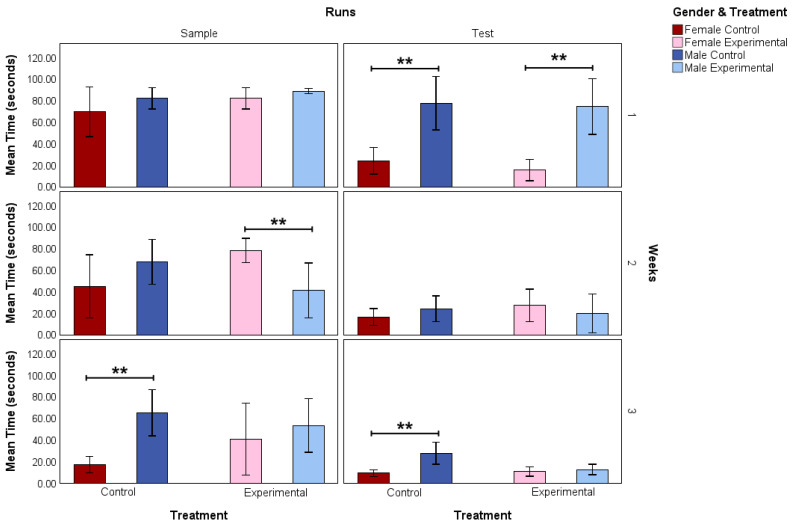
Mean sample run and test run times (seconds) of female control (*n* = 6), male control (*n* = 6), female experimental (*n* = 6), and male experimental (*n* = 6) rats for each of the 3 experimental weeks. Each column represents the 2 different run types and each row represents the 3 experimental weeks. Lines above the bars indicate the pair which was found to have a significant *p*-value. Data are shown as ** *p* < 0.01.

Test results for the effects of gender and treatment within each of the three experimental weeks on the mean sample and run times are shown in Figure 1.

In week 1, the female control mean test run was faster than the male control mean test run (97.5% CI −75.02 to −23.25; *p* = 0.004) while there was no significant difference in mean sample run times. The female experimental mean test run time was also discovered to be significantly lower than the male experimental mean test run time (97.5% CI −79.39 to −27.94; *p* = 0.004). The opposite effect was found in the sample runs during week 2 where the female experimental mean sample run times were higher than the male experimental mean sample run times (97.5% CI 10.40 to 63.31; *p* = 0.004). During the final week, female control mean sample runs were faster than male control mean sample runs (97.5% CI −69.47 to −23.02; *p* = 0.002). Similarly, the female control mean test runs were found to be significantly faster than the male control mean test runs (97.5% CI −30.42 to −10.05; *p* = 0.002).

### 3.2. Body Weight

**Figure 2:**
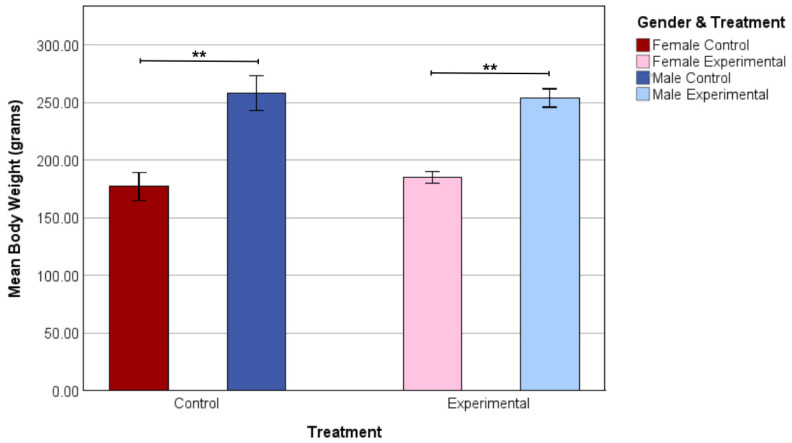
Mean body weight in grams of female control (*n* = 6), male control (*n* = 6), female experimental (*n* = 6), and male experimental (*n* = 6) rats in the final experimental week. Means are represented by bars and lines above the bars indicate a significant difference. Data are shown as ** *p* < 0.01.

The mean body weight of female control rats was significantly lower than the mean body weight of male control rats in the final experimental week (*p* < 0.001). This pattern was also found between the female and male mean body weight in the experimental group of the final week (*p* < 0.001). No mean body weight differences were found between control and experimental groups of female rats and male rats (Figure 2).

### 3.3. Food Intake

**Figure 3:**
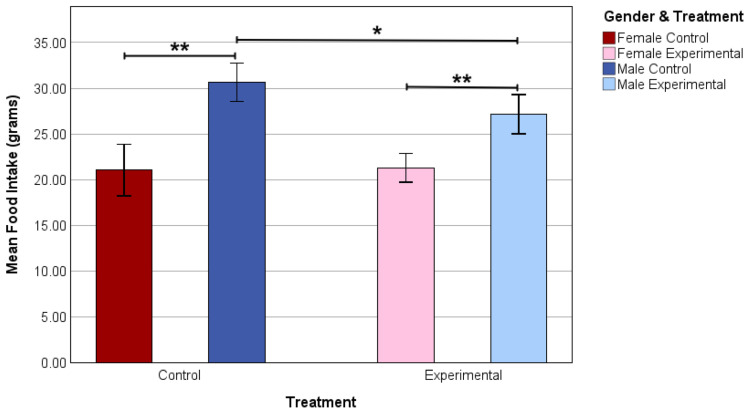
Mean food intake in grams of female control (*n* = 6), male control (*n* = 6), female experimental (*n* = 6), and male experimental (*n* = 6) rats in the final experimental week. Means are represented by bars and lines above the bars indicate a significant difference. Data are shown as * *p* < 0.05 and ** *p* < 0.01.

Mean food intake was significantly different within the final experimental week between the male control group and the male experimental group (*p* = 0.03). No interaction was found between the female control and the female experimental group. In the control group, the mean food intake was significantly higher in male rats compared to female rats (*p* < 0.001). This result was also found in the experimental group between the male and female rats (*p* = 0.001) (Figure 3).

## 4. Discussion

Creatine supplementation has been shown to have a beneficial effect on muscle growth, strength, and recovery as well as improved exercise performance [2]. In addition to these well-known effects, there is evidence of an influence on cognitive processing. The brain is a very metabolically active tissue which contains creatine. Creatine kinase converts creatine into the energy source phosphocreatine, which is primarily stored in the cells to be used as an energy reservoir. The high-energy nature of the phosphate bonds in phosphocreatine allows the compound to be readily available for rapid ATP production in energetically demanding situations [1,5,6].

Using human subjects, several researchers [19,20,21] have observed positive and/or negative or no effects as a result of this type of dietary supplementation. In humans, one test for working memory is backward number recall. Rae et al. [19] demonstrated that creatine supplementation had a significant positive effect on backward digit span using young adult vegetarian subjects. McMorris et al. [20] showed a significant positive effect of creatine supplementation on cognition in the elderly in various tasks except for backward number recall. On the other hand, Rawson et al. [21] reported that creatine supplementation did not improve cognitive function in young adults and suggested that creatine may only be effective in improving cognitive processing in individuals who have initially low basal levels of brain creatine and/or individuals who are either permanently or temporarily suffering from cognitive impairment. Other studies have analyzed the performance of various cognitive tasks under different conditions, such as experimental hypoxia and sleep deprivation [5,6,7]. These reports also provided some conflicting results on the effects of creatine supplementation. 

Because of the few studies conducted on the effects of creatine on spatial working memory in male and female rats, particularly during their natural active period of the circadian cycle, the primary focus of the current research was to investigate this subfield of cognition. Previous research has shown that sex hormones have a significant effect at the transcriptomic level on creatine metabolism within rats’ brains [22,23]. In addition, various studies have demonstrated that epigenetic mechanisms may be involved in producing these effects [24,25]. The results from our MWM assessments indicated that, initially, female rats showed better spatial working memory than male rats. This was evidenced by the fact that both groups of female rats reached the platform during the test trials at a significantly faster rate than their male counterparts (Figure 1). The effects noted in the MWM, however, were unrelated to dietary supplementation, since both control and experimental female rats demonstrated positive working memory. It is noteworthy that, by the second week, all animals of both genders exhibited positive working memory.

In addition to spatial working memory, body weight was analyzed between and across genders. The non-significant difference in body weight between the male rat groups suggests that creatine supplementation did not have an effect on body weight. These findings are in agreement with Young & Young [16], who reported that creatine had no effect on muscle mass, which occurs when there is a net gain of myofibrillar proteins, such as actin and myosin. In addition, a report involving humans found evidence indicating that creatine has no direct anabolic effect on protein synthesis [26]. That study suggests that creatine has a direct effect on athletic performance rather than muscle production. These investigations provide a possible explanation as to why there was no significant difference in body weight between the two male groups in the current research. It should also be noted that no difference in body weight was found between the female control and experimental groups in this study.

While the effects of creatine on body weight were not evident in either gender, there was a significant difference between genders which could be explained by a variety of factors, including the role of sex hormones and age. Sex hormones have been shown to be highly influential in bodily processes. Testosterone promotes male growth by acting on both bone size and mass, while estrogen inhibits female growth in these areas [27,28]. Due to the influence that testosterone has on bone and muscle growth, the male rats would naturally weigh more than the female rats. Age also has a significant influence on the gender difference in body weight. Researchers have found that this gender difference is the most prevalent from the age of four weeks, with maximal growth occurring in the males [28]. These factors could account for the results found between genders in the current study.

To further examine the effects of creatine, food intake was analyzed utilizing the same method as that used to analyze body weight. Unexpectedly, the male rats showed a significant difference in food intake between control and experimental groups, with creatine-supplemented rats consuming less food than control animals. De Moraes et al. [29] found similar results in male exercised vs. sedentary rats when creatine was administered. The exercised rats showed a significant decrease in food intake as compared to all control animals, including sedentary creatine-fed animals. In the current study, all animals had access to an activity wheel in their home cage, which could be considered as a type of exercised animal. The control and experimental female animals displayed no difference in food consumption.

In a similar manner to body weight, sex hormones could potentially play a role in why there was a significant difference in food intake between genders [30,31]. Research conducted on rats found that testosterone increases food intake while estrogen decreases it [28,32]. It has been shown that testosterone plays a pivotal role in appetite. The results of a study analyzing the effects of testosterone on food intake showed that a decrease in testosterone production reduced overall food intake in young male rats [33]. In addition to estrogen decreasing food intake, progesterone has been shown to have a debilitating effect on food intake for females by downregulating glucose production [34].

A limitation of the current study was the use of a small sample size. It is possible that if the number of rats was increased, subtle effects of the supplement could be detected. Bootstrapping has taken this into account in the Statistical Analysis Section of this manuscript. The optimal solution to improve validity is increasing sample sizes, though this is often difficult due to resource and cost constraints. Another limitation would be the age of the rats. If older rats were utilized, there could be a significant effect on body composition, metabolism-related hormones, and energy homeostasis in the two sexes as was noted in Wistar rats by Cognuck et al. [28]. In addition, the MWM may not be sensitive enough to detect subtle cognitive changes. Other behavioral tests such as the Barnes maze or the radial arm maze could be employed.

## 5. Conclusions

The current study adds evidence to the controversy concerning the effects of creatine supplementation on cognitive processing, specifically spatial working memory in rats. Even though gender differences were noted in the MWM, neither gender was affected by the supplement. Similarly, body weight was influenced by gender, but not by creatine supplementation. However, the food intake of experimental male rats increased at a slower rate with continued consumption of the supplement, while female rats showed no difference. Regardless of creatine consumption, female rats consumed less food than male animals.

Because of the popularity of creatine, further research on the effects of this supplement on different mechanisms in the body influencing cognitive processing and appetitive behavior is warranted. In addition, future studies could examine the therapeutic use of creatine supplementation in the treatment of patients identified with brain creatine deficiencies, as well as patients suffering from chronic pathological brain conditions.

## Data Availability

The raw data supporting the conclusions of this article will be made available by the authors on request.
